# Comparative Studies of the Dielectric Properties of Polyester Imide Composite Membranes Containing Hydrophilic and Hydrophobic Mesoporous Silica Particles

**DOI:** 10.3390/ma16010140

**Published:** 2022-12-23

**Authors:** Kuan-Ying Chen, Minsi Yan, Kun-Hao Luo, Yen Wei, Jui-Ming Yeh

**Affiliations:** 1 Department of Chemistry and Center for Nanotechnology, Chung Yuan Christian University, Chung Li District, Taoyuan City 32023, Taiwan; 2 Department of Chemistry, Tsinghua University, Beijing 100084, China

**Keywords:** poly(ester imide), hydrophobic, mesoporous, silica, dielectric

## Abstract

In this paper, comparative studies of hydrophilic and hydrophobic mesoporous silica particles (MSPs) on the dielectric properties of their derivative polyester imide (PEI) composite membranes were investigated. A series of hydrophilic and hydrophobic MSPs were synthesized with the base-catalyzed sol-gel process of TEOS, MTMS, and APTES at a distinctive feeding ratio with a non-surfactant template of D-(-)-Fructose as the pore-forming agent. Subsequently, the MSPs were blended with the diamine of APAB, followed by introducing the dianhydride of TAHQ with mechanical stirring for 24 h. The obtained viscous solution was subsequently coated onto a copper foil, 36 μm in thickness, followed by performing thermal imidization at specifically programmed heating. The dielectric constant of the prepared membranes was found to show an obvious trend: PEI containing hydrophilic MSPs > PEI > PEI containing hydrophobic MSPs. Moreover, the higher the loading of hydrophilic MSPs, the higher the value of the dielectric constant and loss tangent. On the contrary, the higher the loading of hydrophobic MSPs, the lower the value of the dielectric constant with an almost unchanged loss tangent.

## 1. Introduction

In a fully data-centric world populated with telecommunication devices of autonomous vehicles, sensors, robots, and cloud-connected resources, networks need to transfer a greater amount of data than the currently used 4G and 5G systems are capable of handling. To meet the requirements, such as an extraordinary level of data reliability, low latency, low power consumption and massive capacity, devices of the next generation of 5G and 6G wireless communication technologies are expected to use millimeter wave bands (30–300 GHz) [1,2,3,4,5]. These high frequencies imply extreme device densities with small sizes and immunity to interference, thus urging the development of new materials with unique properties and structures with unorthodox designs [6,7].

The polyimide membrane is a high-performance polymer with good dielectric properties, thermal stability, and chemical resistance [8,9]. It is widely used in the electronic industry. In recent years, with the vigorous development of mobile communication devices, the demand for the size reduction in integrated circuits has driven the advanced process line width of 7, 5, and 3 nm [10]. Low dielectric interlayer materials not only reduce the current leakage of integrated circuits but also improve the capacitance effect between wires, RC time delay, cross-talk, heat generation, and power dissipation in the new generation of high-density integrated circuits [11,12]. Commercially available polyimide has a dielectric constant between 3.1 and 3.6 [13], which is not enough for future development. Hence, extensive research on polyimides with low dielectric constant materials was reported [14,15,16,17].

There are two main approaches to reducing the dielectric constant of the polyimide polymer, both aiming at reducing the polarizability of the material: (1) introducing a fluorine atom or lipid ring structure [18,19] and (2) increasing the free volume of the structural skeleton or introducing air [20,21]. The dielectric constant of air is 1 [22], which is intrinsically low in comparison to water, which is approximately 80 [23]. Introducing air into the polyimide structure effectively reduces the dielectric constant of the overall material. This porous structure could be achieved through the direct introduction of air, such as polyimide aerogels, which requires a time-consuming solvent exchange process and the precise conditioning of a drying method. Another method is compositing with other porous materials. Mesoporous silica has gained much attention in recent research as a molecular sieve and has already been explored in organic and inorganic composites to achieve low dielectric constant materials [24]. The preparation of mesoporous silica could be further differentiated into silica with regular porous structures and irregular porous structures. Stucky et al. and Kresge et al. both demonstrated the formation and modification of MCM-41 silica using a surfactant as a template, which requires high-temperature calcination to remove the temperate [25,26]. This method is industrially unfavorable due to its high energy requirement and the nature of processing complications. To form silica with irregular porosity, Yen et al. conducted extensive research on a nonsufactant template method forsol-gel process using a hydroxy-carboxylic acid as a template [27,28,29]. In the same period, Yen et al. proposed a novel one-step method for enzyme encapsulation in mesoporous material using D-glucose as a template [30]. Furthermore, according to the study [31], the ester segment functional group is the dominant factor for low water uptake; thus, the PEI polymer system is a promising candidate as a novel dielectric substrate material for use in the next generation of high-performance flexible printed circuit boards operating at higher frequency (10 GHz).

Therefore, in this study, we attempted to prepare a PEI membrane with lower dielectric properties (e.g., the dielectric constant and loss tangent) by incorporating hydrophobic MSPs, which could effectively decrease the dielectric constant of the PEI membrane by introducing a large amount of air into the porous channels and significantly decreasing the absorption amount of moisture by the MSPs. The hydrophobic MSPs were synthesized via a base-catalyzed sol-gel reaction with D-(-)-Fructose as a non-surfactant template. Subsequently, the hydrophobic inorganic MSPs were introduced into the organic PEI membrane with a lower loss tangent to prepare the organic-inorganic hybrid membranes with a lower dielectric constant and loss tangent simultaneously. Moreover, the hydrophilic MSPs were also synthesized as a control experiment. The characterizations of the hydrophilic and hydrophobic MSPs were investigated and compared using FTIR, ^13^C-NMR, ^29^Si-NMR spectra, and EDS. The physical properties of the hydrophilic and hydrophobic MSPs were studied and compared using SEM, TEM, CA, TGA, and BET. Subsequently, comparative studies for the effect of hydrophilic and hydrophobic MSPs on the dielectric properties of the PEI and its corresponding composite membranes were systematically investigated. The mechanical strength, thermal properties, and surface wettability of the PEI and its corresponding composite membranes were also investigated using the tensile test, TGA, and CA, respectively.

## 2. Experiment

### 2.1. Materials

*p*-Phenylene bis(trimellitate) dianhydride (TAHQ) and 4-Aminophenyl-4-aminobenzoate (APAB) were both obtained from SHIFENG TECHNOLOGY CO., LTD (Tainan City, Taiwan) and were dried in an oven overnight before use. Tetraethyl orthosilicate (TEOS), trimethoxymethylsilane (MTMS), and ammonium hydroxide, (ACS reagent, 28.0–30.0% NH_3_ basis) were purchased from Sigma-Aldrich (St. Louis, MO, USA) and used as received. Triethoxysilane (3-Aminopropyl) (APTES) and D-(-)-Fructose, both of 99% purity, were purchased from Sigma-Aldrich and used as received. N-Methyl-2-pyrrolidone (NMP) was purchased from Macron and stored in a container with molecular sieves overnight before use. Ethanol was purchased from J.T. Baker and used as received.

### 2.2. Instrumentation

All samples were characterized through an attenuated total reflectance FT-IR in the form of potassium bromide (KBr) powder-pressed pellets. Both the ^13^C and ^29^Si MAS solid-state NMR experiments were performed on a 400 MHz solid-state NMR spectrometer. ^13^C MAS NMR spectra were obtained at 100.63 MHz with 7 kHz applying 90° pulses and 2.0 s pulse delays. ^29^Si MAS NMR spectra were recorded at 79.49 MHz applying 90° pulses, 300 s pulse delays, and 5.0 ms of contact time, with samples in 5.0 mm zirconia rotors spinning at 7 kHz. The surface morphologies of the superhydrophobic samples were observed by using SEM (JOEL JSM-7600F). The silica dispersion was detected using energy dispersive spectroscopy (EDS) (Oxford xmax 80). The crystallographic structure of the polymer was studied with the X-ray diffraction (XRD) pattern on a Bruker D8 Advance Eco instrument using Cu Kα radiation (λ = 1.5418 nm, 40 kV, 25 mA) at a scanning rate of 2° min^−1^. Thermogravimetric analysis (TGA) was used to analyze the thermal stability and moisture content of the sample under N_2_ at constant pressure, with a temperature elevation rate of 10 °C/min, from 30 °C to 800 °C. The tensile strength, elongation at break, and Young’s modulus were tested and recorded using a Hung Ta HT-9102 tensile test machine. Brunauer-Emmett-Teller (BET) data were obtained by performing nitrogen adsorption/desorption isotherms and accelerated surface area porosity analysis on Micromeritics ASAP-2010. The hydrophilicity of the sample was tested based on the contact angle (CA) of the water droplets on the prepared sample. The results were measured with a contact angle FTA125 instrument. The microstructure and surface morphology were studied using a transmission electron microscope (TEM) and scanning electron microscope (SEM), respectively. Lastly, all prepared samples were tested under impedance spectroscopy at 10 GHz with an AET Microwave Dielectrometer to measure their loss tangent and capacitance. The dielectric constant κ was calculated from the equation listed below, where C is the capacitance, ε_0_ is the absolute dielectric constant, A is the component electrode surface area, and d is the thickness.

$$C=\frac{\kappa \varepsilon_{0}A}{d}$$

### 2.3. Synthesis of the Hydrophilic and Hydrophobic MSPs

**Scheme 1:**
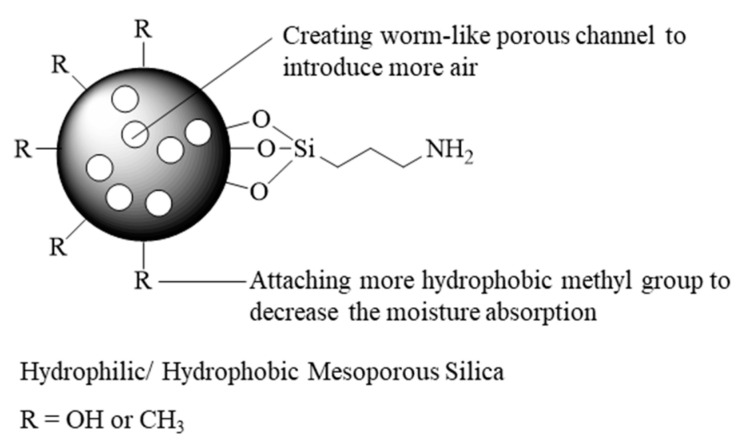
Designed concept of synthesizing hydrophobic mesoporous silica particles (MSPs), with hydrophilic primary and hydrophobic methyl group as well as worm-like porous channels, functioning as fillers that decrease the dielectric properties of the polyester imide (PEI) composite membrane.

**Scheme 2:**
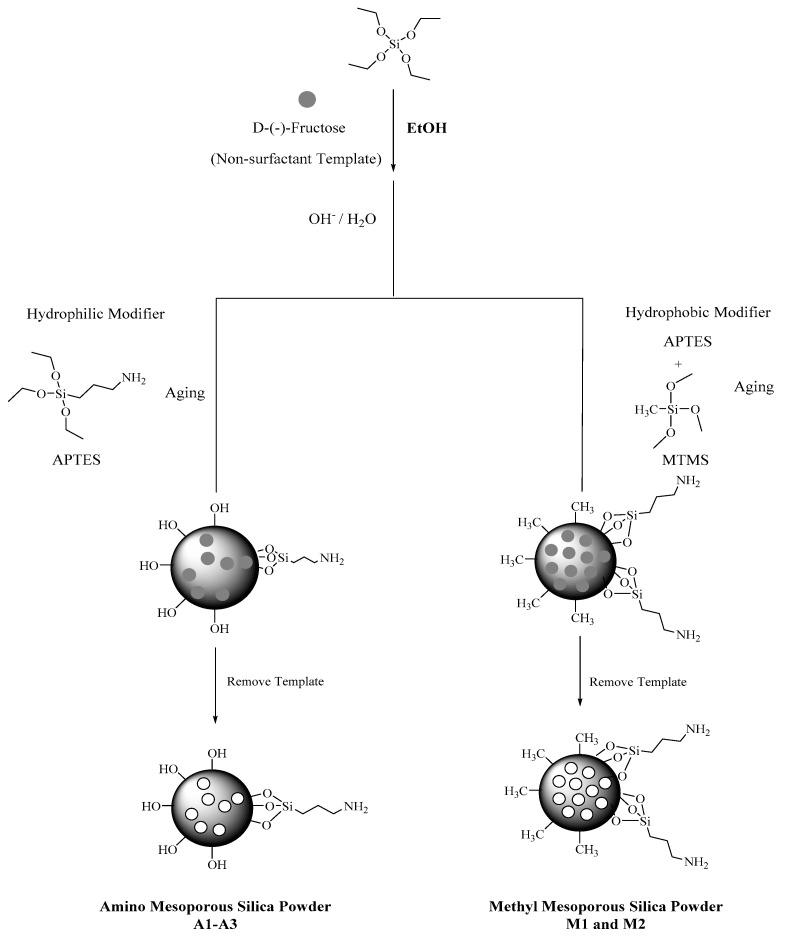
Flowchart for the synthesis of the (**a**) hydrophilic amino-modified mesoporous silica particles (MSPs) and the (**b**) hydrophobic methyl-modified MSPs through the base-catalyzed sol-gel route with D-(-)-Fructose as a non-surfactant template.

The fundamental design concept to synthesize the MSPs was to perform the base-catalyzed sol-gel process and to create the worm-like porous channels through a non-surfactant template route, as shown in Scheme 1. To prepare the hydrophilic MSPs, the base-catalyzed sol-gel reactions of TEOS and APTES (with a hydrophilic -NH_2_) at a specific feeding ratio in the presence of the non-surfactant template of D-(-)-Fructose as the pore-forming agent was used. Moreover, to prepare the hydrophobic MSPs, the sol-gel reactions of TEOS, APTES (with a hydrophilic -NH_2_), and MTMS (with a hydrophobic -CH_3_) at a specific feeding ratio in the presence of D-(-)-Fructose were also employed. The detailed formulations to synthesize the hydrophilic MSPs (A1, A2, and A3) and hydrophobic MSPs (M1 and M2) are shown in Table 1. The flowchart of synthesizing the hydrophilic MSPs (A1–A3) and hydrophobic MSPs (M1 and M2) via the base-catalyzed sol-gel reaction in the presence of D-(-)-Fructose is illustrated in Scheme 2. The typical representative procedure to synthesize the hydrophilic and hydrophobic MSPs was as follows: Based on the formulation presented in Table 1, an appropriate amount of D-(-)-Fructose was poured into a double-layer beaker containing 200 mL of ethanol and 100 mL of deionized water with magnetic stirring at 35 °C. Subsequently, 9.0 mL of concentrated ammonia was dropped into the previous beaker while stirring. Three sol-gel precursors (i.e., TEOS, APTES, or MTMS) at specific feeding ratios were injected into the beaker with a syringe with magnetic stirring for 48 h. The synthesized products were collected by centrifuging the solution and then ultrasonically washing the collected paste with ethanol and water 3 and 10 times, respectively, to remove the D-(-)-Fructose. The washing of the products was followed by the freeze-drying procedure to obtain the final hydrophilic MSPs (A1, A2, and A3) and hydrophobic MSPs (M1 and M2).

### 2.4. Preparation of PEI Composite Membranes Containing Hydrophilic and Hydrophobic MSPs

To prepare the PEI composite membrane, distinctive MSPs were incorporated into the polyester imide at specific feeding ratios; the detailed formulations for the preparation of the composite membranes are shown in Table 2. A typical procedure to prepare the composite membrane was as follows: First, a suitable amount of diamine monomer was dissolved in a 3-neck rounded flask with 40 g of NMP at room temperature under a nitrogen atmosphere. Subsequently, different ratios of MSPs were then stirred into the mixture. A specific amount of dianhydride was then introduced with magnetic stirring for 24 h. As the reaction time increased, the viscosity of the MSP-containing mixing solution was gradually increased to produce the poly(ester amic acid) (PEAA)/MSP composites, followed by coating on top of a copper foil with a blade coater.

**Scheme 3:**
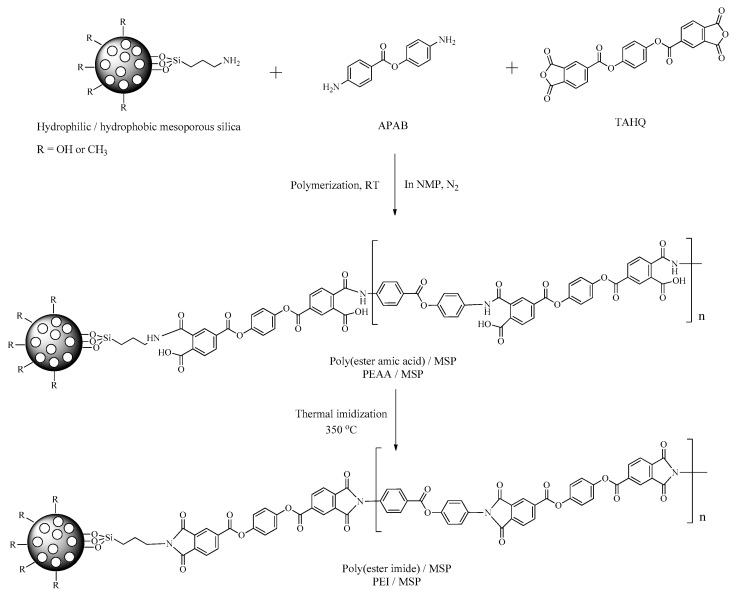
Flowchart of the preparation of the PEI composite membranes containing five distinctive MSPs via thermal imidization.

Subsequently, the prepared mixing solution was followed by performing a thermal imidization reaction to form a polyester imide (PEI). The programmed heating conditions were as follows: (150 °C, 5 min), (200 °C, 5 min), (250 °C, 5 min), (300 °C, 30 min), and (350 °C, 30 min). After immersing the PEI-coated copper foil in an acid etching solution for 30 min, the final PEI membrane was then obtained, which was 36 μm in thickness. The flowchart for the preparation of the PEI composite membranes containing hydrophilic and hydrophobic MSPs is shown in Scheme 3.

## 3. Results and Discussion

In this section, the characterization of the prepared materials was classified into two parts: the inorganic MSPs and organic-inorganic PEI composite membranes.

### 3.1. Characterization of the Hydrophilic and Hydrophobic MSPs

#### 3.1.1. FTIR Spectra

**Figure 1:**
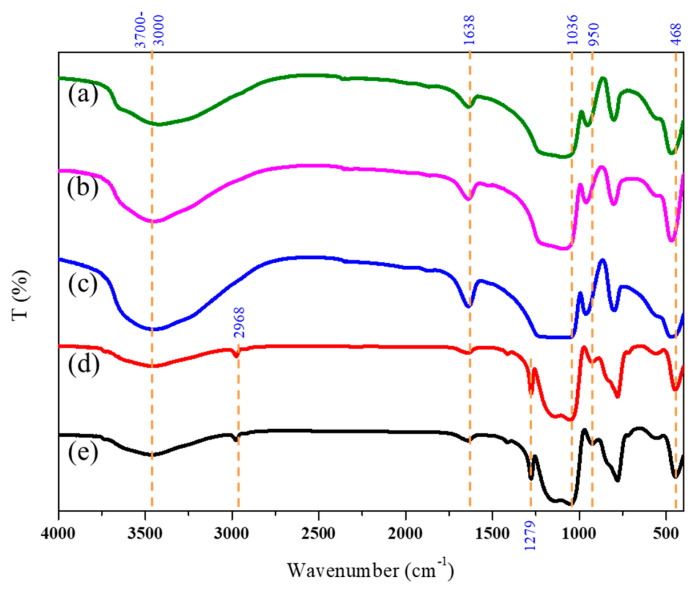
ATR-FTIR spectra of the hydrophilic (**a**) A1, (**b**) A2, and (**c**) A3 MSPs, as well as the hydrophobic (**d**) M1 and (**e**) M2 MSPs.

Figure 1 shows the representative FTIR spectra of the synthesized distinctive MSPs (i.e., A1, A2, A3, M1, and M2). First, the characteristic peak for the symmetrical absorption peak of Si-O-Si appeared at the wavenumber of 950 cm^−1^. Moreover, the characteristic peak for the asymmetric absorption of Si-O-Si appeared at the position of 1036 cm^−1^ [32]. A broad-band peak of Si-OH was observed from 3000 to 3700 cm^−1^. The characteristic peak that appeared at the position of 1638 cm^−1^ was assigned to be the bending of a hydrophilic primary amine -NH_2_ [33], which confirmed the participation of APTES in the sol-gel reactions for the A1, A2, and A3 MSPs. On the other hand, for the characterization of the M1 and M2 hydrophobic MSPs, the characteristic peak for the asymmetric absorption of Si-CH_3_ and hydrophobic -CH_3_ was observed at the positions of 1279 and 2968 cm^−1^ [34], respectively, which confirmed the participation of MTMS in the sol-gel reactions of M1 and M2. Furthermore, it is worth noting that the bonding between MTMS and APTES on the surface is triple bonding. Certain studies [35,36,37] have mentioned that in the spectra of FT-IR, the peaks at 1050–1150 cm^−1^ are characteristic of C-O-C, Si-O-C, and Si-O-Si stretching. This was observed in this study in Figure 1.

#### 3.1.2. Solid-State ^29^Si-NMR Spectra

**Figure 2:**
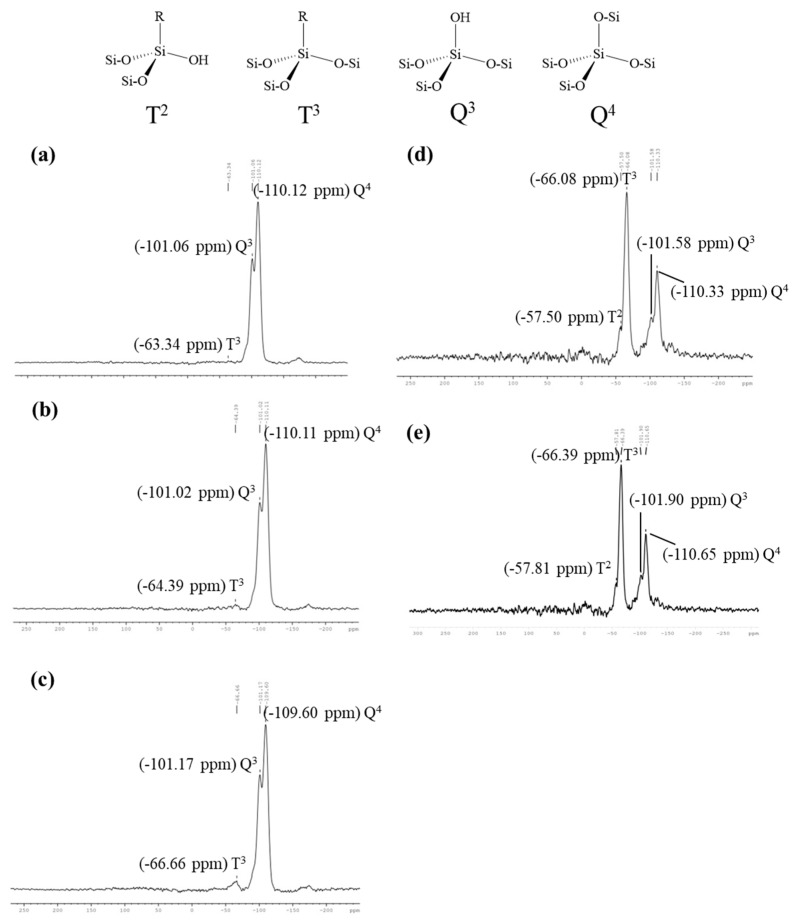
Solid-state 29Si-NMR spectra of the hydrophilic (**a**) A1, (**b**) A2, and (**c**) A3 MSPs, as well as the hydrophobic (**d**) M1 and (**e**) M2 MSPs.

Figure 2 shows the solid-state ^29^Si-NMR spectra of the hydrophilic and hydrophobic MSPs. For example, Figure 2a–c exhibits the solid-state ^29^Si-NMR spectra of the hydrophilic A1, A2, and A3 MSPs. The hydrophilic characteristics of A1~A3 were attributed to the hydrophilic group of the primary amine from APTES reacting with TEOS via the base-catalyzed sol-gel reactions. For the characterization of the hydrophilic MSPs, the T3 signal of APTES was observed at the chemical shift of ~−60 ppm. Moreover, the Q^3^ and Q^4^ signals of TEOS were observed at chemical shifts of −101 ppm and −110 ppm [38,39], respectively. However, the signal intensity and integral area of the Q series were found to be larger than those of the T series, which may be attributed to the higher feeding molar ratio of TEOS than that of APTES. On the other hand, Figure 2d,e are the solid-state silicon NMR spectra of the hydrophobic M1 and M2 MSPs. The hydrophobic characteristics of M1 and M2 were attributed to the hydrophobic -CH_3_ from MTMS reacting with TEOS via the base-catalyzed sol-gel reactions. However, the signal intensity and integral area of the T series were found to be larger than those of the Q series, which may be attributed to the higher feeding molar ratio of APTES and MTMS than that of TEOS.

#### 3.1.3. Solid-State ^13^C-NMR Spectra

**Figure 3:**
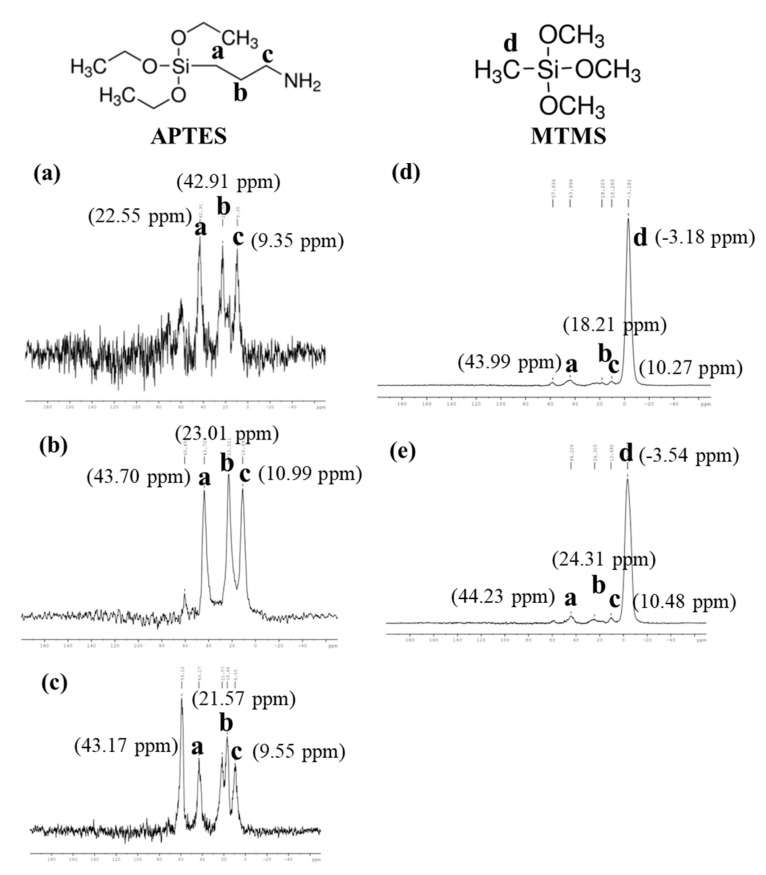
Solid-state 13C-NMR spectra of the hydrophilic (**a**) A1, (**b**) A2, and (**c**) A3 MSPs, as well as the hydrophobic (**d**) M1 and (**e**) M2 MSPs.

Figure 3 reveals the solid-state ^13^C-NMR spectra of the hydrophilic and hydrophobic MSPs. Figure 3a–c shows the hydrophilic A1, A2, and A3 MSPs modified by APTES. In these hydrophilic MSPs, the chemical shifts of carbon for **a**, **b** and **c** were found to appear at the positions at approximately 43 ppm, 22 ppm, and 10 ppm [40], respectively, which indicated the participation of APTES in the sol-gel reactions of A1, A2, and A3. On the other hand, the hydrophobic M1 and M2 MSPs modified by MTMS were prepared by incorporating a small amount of hydrophilic APTES and a large amount of hydrophobic MTMS. Figure 3d,e shows the solid-state ^13^C-NMR spectra for the hydrophobic M1 and M2 MSPs modified by APTES and MTMS. In these MSPs, the chemical shifts of carbon for a, b and c were found to appear at positions at approximately 43 ppm, 22 ppm, and 10 ppm [40], respectively, which indicated the participation of APTES in the sol-gel reactions of M1 and M2. Moreover, the chemical shift of carbon for **d** was found to appear at the position of −3.5 ppm, which confirmed the participation of MTMS in the sol-gel reactions of M1 and M2.

#### 3.1.4. Energy Dispersive Spectroscopy (EDS)

The elemental analysis of the EDS spectra for the three hydrophilic MSPs (A1, A2, and A3) confirmed that the EDS data of A3 exhibited the highest N element content of 1.29 wt-%, and A1 revealed the lowest content of 0.72 wt-%, which was consistent with the highest and lowest initial feeding ratios of APTES in A3 and A1, respectively, as summarized in Table 1.

### 3.2. Physical Properties of MSPs

#### 3.2.1. Surface Morphology of the Hydrophilic and Hydrophobic MSPs (SEM)

**Figure 4:**
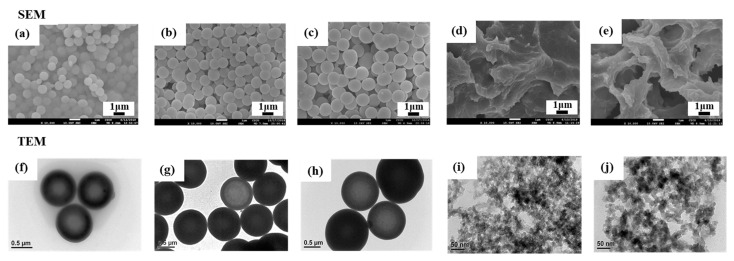
SEM for (**a**) A1, (**b**) A2, (**c**) A3, (**d**) M1, and (**e**) M2, as well as the transmission images of the TEM for (**f**) A1, (**g**) A2, (**h**) A3, (**i**) M1, and (**j**) M2.

The SEM images of the surface morphologies for the hydrophilic MSPs (A1, A2, and A3) were found to be spherical, as shown in Figure 4a–c. It should be noted that with the increase in APTES content in the hydrophilic MSPs, an increase in the diameter of silica particles occurred. For example, the average hydrophilic MSP diameters of A1, A2, and A3 were ~800 nm, ~1000 nm, and ~1200 nm, respectively. It indicated that a higher loading of amino-silane may slightly increase the diameter of the corresponding hydrophilic MSPs. This conclusion is consistent with the previous publication reported by Krysztafkiewicz et al. [41]. At the same time, the surface morphologies of the M1 and M2 MSPs modified by the fixed feeding ratio of MTMS6 with different dosages of the pore-forming agent were found to be irregular in shape, as shown in Figure 4d,e.

#### 3.2.2. Transmission Morphology of the Hydrophilic and Hydrophobic MSPs (TEM)

This section may be divided into subheadings. It should provide a concise and precise description of the experimental results, their interpretation, as well as the experimental conclusions that could be drawn. For the TEM observation, the MSP samples were first dispersed in ethanol, followed by dropping on the copper mesh for the observation of the TEM image; the darker and brighter regions of the TEM imagery indicated that the thickness of the silica particle wall was thicker and thinner, respectively. For example, the TEM image of hydrophilic MSPs (A1, A2, and A3) is shown in Figure 4f, Figure 4g, and Figure 4h, respectively. Moreover, the TEM images of the M1 and M2 MSPs were found to exhibit a mesoporous structure, as shown in Figure 4i and Figure 4j, respectively.

#### 3.2.3. Surface Wettability

**Figure 5:**
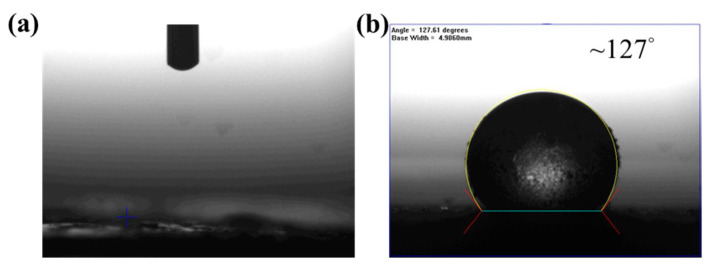
Contact angle of the water droplets for the (**a**) hydrophilic MSPs (A1, A2, and A3). (un-detectable) and the (**b**) hydrophobic MSPs (M1 and M2).

In these studies, the hydrophilic MSPs (A1, A2, A3) and hydrophobic MSPs (M1 and M2) in powder form were first fabricated into the shape of a powder-pressed pellet before performing the CA measurements of the water droplets. It should be noted that the CA of three hydrophilic MSPs (i.e., A1, A2, and A3) could not be detected, as shown in Figure 5a, which might be attributed to the super-hydrophilic characteristics of the prepared MSPs modified with a primary amine group. Moreover, the CAs of the two hydrophobic MSPs (i.e., M1 and M2) were both found to be ~127°, as shown in Figure 5b. This indicated that the incorporation of MTMS into the MSPs may effectively increase the CA of the hydrophilic MSPs, which was attributed to the introduction of the hydrophobic -CH_3_ group. All the data of the CA measurements of the water droplets for the MSPs are summarized in Table 1.

#### 3.2.4. Determination of the Moisture Absorption of the MSPs with TGA

**Figure 6:**
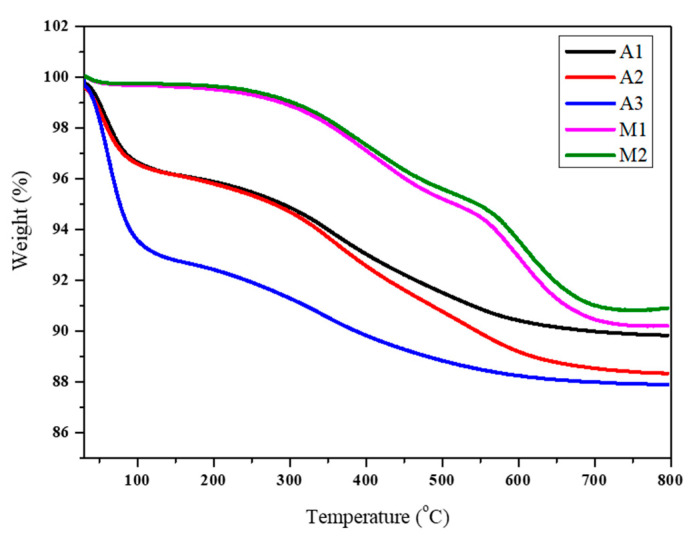
TGA curves of the hydrophilic MSPs (A1, A2, and A3) and the hydrophobic MSPs (M1 and M2).

The TGA of the five distinctive hydrophilic and hydrophobic MSPs was operated at temperatures ranging between 30 °C and 800 °C in the air at a heating rate of 10 °C/min, as shown in Figure 6. In this study, the moisture absorption of MSPs was defined as the weight loss at 200 °C, and the data are summarized in Table 1. The moisture absorptions of the hydrophilic MSPs (A1, A2, and A3) were estimated at weight losses of 4.15, 4.24, and 7.68 wt-%, respectively, as shown in Figure 6. It indicated that the A3 sample revealed the highest hygroscopicity. The significant weight loss observed at 100 °C is the water content from the A3 MSPs. Compared to A1 and A2, A3 possessed the highest content of hydrophilic functional groups. Hence, it absorbed more moisture at RT.

On the other hand, the moisture absorptions of the hydrophobic MSPs (M1 and M2) were estimated at weight losses of 0.58 and 0.45 wt-%, respectively, as shown in Figure 6. It demonstrated that M2 exhibited the lowest hygroscopicity. These results confirmed that the MSP modified by MTMS could effectively improve the corresponding hydrophobicity compared to that of MSPs modified by APTES. Moreover, the lower hygroscopicity of M2 as compared to that of M1 may be attributed to the hydrophobic -CH_3_ attached to the higher specific surface area of the MSP resulting from the higher dosage of the pore-forming agent. It should be noted that the trend of the MSP’s moisture absorption determined with the TGA was consistent with the previous studies of the CA measurements of the water droplets. All the data on the moisture absorptions of the MSPs are summarized in Table 1.

#### 3.2.5. BET Analysis of the MSPs

**Figure 7:**
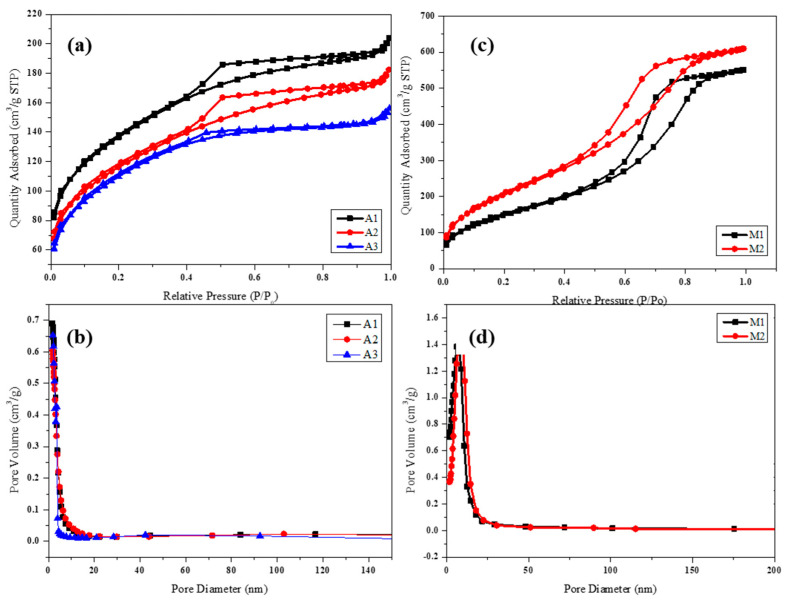
BET analysis of the series of MSPs. (**a**) The N_2_ adsorption-desorption isotherm as well as (**b**) the pore size distribution curves for the hydrophilic MSPs (A1, A2 and A3). (**c**) The N_2_ adsorption-desorption isotherm and (**d**) pore size distribution curves of the hydrophobic MSPs (M1 and M2).

Figure 7a,b exhibit the N_2_ adsorption-desorption isotherm and pore diameter distribution curves of the hydrophilic MSPs, respectively. It should be noted that a higher loading of APTES in the MSPs resulted in a smaller specific surface area. This indicated that the trend of the specific surface area for the hydrophilic MSPs was A1 (469 ± 12 m^2^/g) > A2 (402 ± 21 m^2^/g) > A3 (384 ± 14 m^2^/g). The slightly decreasing surface area of the MSPs with higher APTES may be attributed to the longer chains of primary amines of APTES existing inside the mesoporous channel after the removal of D-(-)-Fructose. The average pore size distributions of the A1, A2, and A3 MSPs were 2.9, 3.0, and 2.7 nm, respectively.

Figure 7c,d exhibit the N_2_ adsorption-desorption isotherm and pore diameter distribution curves of the hydrophobic MSPs, respectively. It should be noted that a higher loading of MTMS in the MSPs resulted in a larger specific surface area. This indicated that the specific surface area for the hydrophobic MSPs was M2 (771 ± 14 m^2^/g) > M1 (548 ± 12 m^2^/g). The specific surface area of the hydrophobic M2 MSP compared to that of M1 was attributed to the higher loading of the pore-forming agent, which is consistent with a previous report [42]. The average pore size distributions of the M1 and M2 MSPs were found to be ~4.9 and ~6.2 nm, respectively. All the data of the BET analysis of the surface areas, pore volumes, and average pore diameters for the five distinctive MSPs are summarized in Table 1.

### 3.3. Characterization of the MSP-Based PEI Composite Membranes

#### 3.3.1. ATR-FTIR Spectra

**Figure 8:**
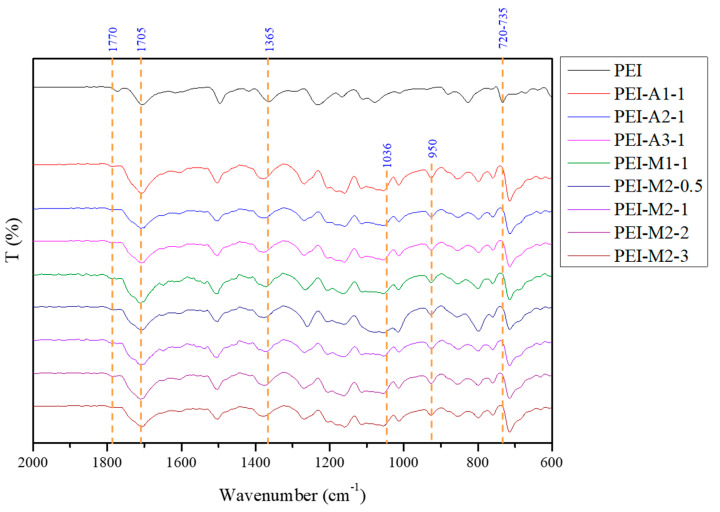
ATR-FTIR spectra of the polyester imide (PEI) and its corresponding composite membranes containing hydrophilic MSPs (A1, A2, and A3) and hydrophobic MSPs (M1 and M2).

In this study, the characterizations of the MSP-based PEI composite membranes were measured with ATR-FTIR, as shown in Figure 8. It should be noted that FTIR spectra of all composite membranes revealed the characteristic peaks of Si-O-Si groups except that of the neat PEI membrane. For example, the characteristic symmetric and asymmetric absorption peaks of Si-O-Si were found to appear at wavenumbers of 950 cm^−1^ and 1036 cm^−1^, respectively [32]. On the other hand, the characteristic peak that appeared at the position of 735 cm^−1^ was attributed to the formation of the imide ring, which indicated the successful preparation of the PEI with thermal imidization [42]. However, a redshift was clearly observed for the PEI/MSP composite at this characteristic peak. According to Okada et al. [43], the reason for the possible redshift in the spectrum of the imide ring functional groups may be that the PEI is an ordered molecular chain, while silica has a network structure. When silica and imide ring are combined, the intervention of steric barriers deforms the orderly arrangement into an out-of-order one, thus producing a redshift phenomenon. Moreover, the characteristic peaks that appeared at the position of 1705 cm^−1^ and 1770 cm^−1^ were assigned to be the symmetric stretching of the imide ring and asymmetric C=O stretching of the imine ring, respectively [37].

#### 3.3.2. XRD Pattern

**Figure 9:**
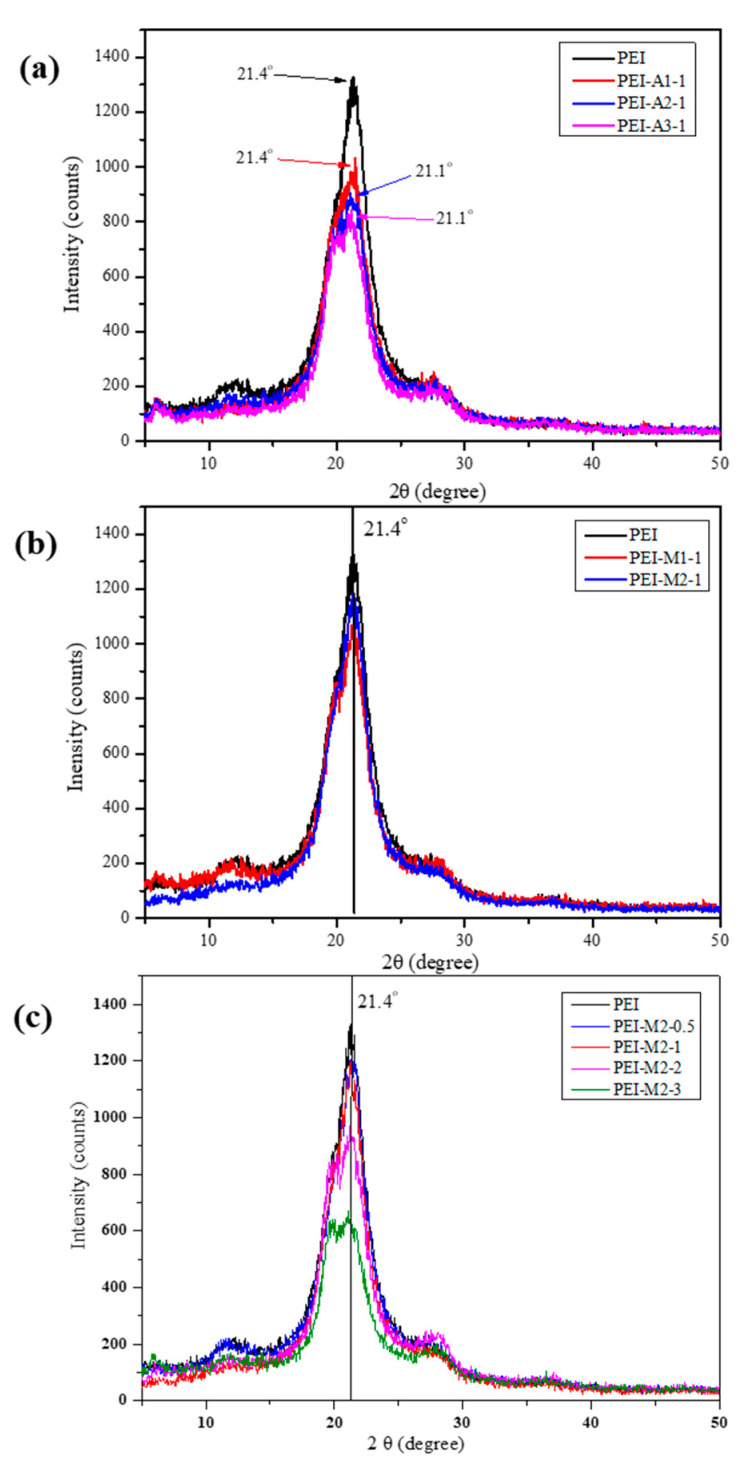
XRD curves of the PEI and its corresponding composite membranes containing the (**a**) hydrophobic MSPs (A1, A2, and A3), (**b**) hydrophobic M1 MSP, and (**c**) hydrophobic M2 MSP.

In this characterization, the crystal behavior of the PEI membrane in the presence of the hydrophilic and hydrophobic MSPs was studied using the XRD pattern, as shown in Figure 9. The Bragg angle of 2θ = ~22° indicated that all prepared samples were of amorphous materials, attributed to the presence of the MSPs [44]. The orderly arrangement of the PEI molecules was disrupted in the presence of the MSPs, therefore leading to a decrease in the crystallinity of the PEI membranes. The full width at half maximum (denoted by FWHM) of the XRD profile was sensitive to the variation in the microstructure and stress-strain accumulation in the material. The FWHM value is inversely proportional to the crystallite size (Scherrer’s formula), meaning the broader the peak, the smaller the crystallite dimension [45]. For example, by incorporating 1 wt-% A1, A2, and A3 MSPs, the FWHM value of the PEI increased from 3.2 to 3.4, 3.5, and 3.5, respectively, indicating that the incorporation of the A1, A2, and A3 MSPs into the PEI may decrease the crystallite size of the PEI membrane, as shown in Figure 9a and Table 2. On the other hand, incorporating 1 wt-% of the M1 and M2 MSPs, the FWHM value of the PEI increased from 3.2 to 3.3 and 3.3, respectively. It indicated that the PEI composite membranes incorporating 1 wt-% hydrophobic MSPs exhibited a lower FWHM value than that of 1 wt-% hydrophilic MSPs. Moreover, the higher loading of M2 in the PEI composite membranes may result in a higher FWHM value, reflecting a decrease in the crystallite size of the PEI membrane.

### 3.4. Physical Properties of MSP-Based PEI Composite Membranes

#### 3.4.1. Dielectric Properties

In this study, the high-frequency dielectric measurement used the resonant cavity with impedance spectroscopy to measure the microwave dielectric of the substance, and the measured frequency used was 10 GHz. The dielectric constant and loss tangent of the PEI composite membranes containing the MSPs was measured in the 10 GHz frequency band, as summarized in Table 2. For example, the dielectric constant (D_k_) and loss tangent (D_f_) of the neat PEI were 3.27 and 0.007, respectively. By incorporating 1 wt-% hydrophilic A1, A2, and A3 MSPs, the D_k_ of the corresponding PEI composite membrane was found to increase up to 3.43, 3.46, and 3.92, respectively. The D_f_ increased up to 0.011, 0.012, and 0.015, respectively. The obvious increase in the dielectric constant and loss tangent in the PEI by incorporation of 1 wt-% hydrophilic MSPs may be attributed to two possible reasons: the higher moisture absorption and smaller crystallite size of the PEI membrane. The weight percentage of the moisture absorption of the PEI and corresponding composite membranes of PEI-A1-1, PEI-A2-1, and PEI-A3-1 were estimated to be 0.73, 0.95, 0.98, and 1.18 wt-%, respectively, based on Figure 6 and Table 2. According to the previous publications, the D_k_ and D_f_ values of H_2_O at room temperature tested under 10 GHz were reported to be ~80 and ~0.47 [46]. Therefore, the PEI composite membranes with higher moisture absorption capabilities may reveal higher D_k_ and D_f_ values.

On the other hand, by incorporating 1 wt-% hydrophobic M1 and M2 MSPs, the D_k_ of the corresponding PEI composite membrane was found to be suppressed to 3.24 and 3.18, respectively. In comparison, the D_f_ retained roughly the same values as the PEI alone, which were 0.009 and 0.008, respectively. The weight percentage of the moisture absorptions of the PEI-M1-1 and PEI-M2-1 PEI composite membranes were estimated to be 0.69 and 0.52 wt-%, respectively, based on the TGA investigation. The decrease of the D_k_ may be attributed to two possible reasons: (1) the low moisture absorption of the PEI or (2) the high surface area of silica with worm-like pore channels. First, the low moisture absorption of the PEI membrane may be related to the incorporation of the hydrophobic MSPs (M1 and M2). Second, the higher surface area may lead more air (D_k_ of air = ~1) into the porous channel of the hydrophobic MSP1s, reflecting a decrease in the D_k_ of the PEI membrane.

For the studies of the dielectric properties of the PEI composite membrane containing the M2 MSP at different loadings, the D_k_ of the PEI composite membrane containing 1 wt-%, 2 wt-%, and 3 wt-% of M2 were found to be 3.18, 2.97, and 2.93, respectively. On the other hand, the D_f_ of the PEI composite membrane containing 1 wt-%, 2 wt-%, and 3 wt-% of M2 were found to be 0.008, 0.008, and 0.009, respectively.

#### 3.4.2. Mechanical Strength

The tensile modulus and elongation at the break of the PEI membrane were measured using the tensile test, according to ASTM D882 [47], and were found to be 11.26 MPa and 1.48%, respectively. By incorporating 1 wt-% hydrophilic MSPs, the tensile moduli of the PEI composite membranes containing 1 wt-% of A1, A2, and A3 were found to decrease to 4.03, 4.80, and 5.48 MPa, respectively. Moreover, the elongations at the break of the PEI composite membranes containing 1 wt-% of A1, A2, and A3 were found to increase to 2.15%, 2.52%, and 2.75%, respectively.

On the other hand, by incorporating 1 wt-% hydrophobic MSPs, the tensile moduli of the PEI composite membranes containing 1 wt-% of M1 and M2 were found to significantly increase to 10.22 MPa, and 16.23 MPa, respectively. At the same time, the elongations at the break of the PEI composite membranes containing 1 wt-% of M1 and M2 were found with noticeable reductions, which were 1.64% and 1.20%, respectively. It indicated that the incorporation of the hydrophobic M2 MSP with a higher surface area of 771 m^2^/g promoted the mechanical strength and decreased the elongation at the break of the PEI membrane simultaneously as compared to the hydrophobic M1 MSP with the lower surface area of 548 m^2^/g.

Eventually, by incorporating the M2 hydrophobic MSP at different feeding ratios of 1 wt-%, 2 wt-%, and 3 wt-% in the PEI membrane, the mechanical strengths of the neat PEI were found to be 16.23, 18.95, and 6.68 MPa of the PEI composites, respectively. Moreover, the elongations at the break of the neat PEI composites decreased from 1.20% to 1.19% and then escalated to 1.65%. It implied that, by comparing the 1 and 2 wt-% loading of M2, the higher the loading of M2 in the PEI membrane, the higher the mechanical strength and the lower the elongation at the break of the composite membranes. The decrease of the mechanical strength and increase in the elongation at the break of the PEI composite membrane at 3 wt-% of M2 loading may be attributed to the M2 particle aggregation that occurred at the higher loading of the MSPs. All test measurements are collectively listed in Table 2.

It is speculated that there was physical crosslinking caused by the strong interfacial interaction between the PEI molecules and MSPs. This may be the core reason for the strengthening effects observed. Within an appropriate range of crosslinking density, both a strengthening and toughening effect was observed. However, excessive incorporation would lead to agglomerates, which create defects within the polymetric structure, hence decreasing the overall mechanical properties. Although it is widely believed in a rubber/polymer system, the strengthening effect is a result of void formation in rubber particles under stress [48], it is unlikely to be what was found in this PEI/MSP composite system since the Si-O bond is too strong to be broken before the failure of the composite.

#### 3.4.3. Moisture Absorption and Thermal Stability Determined with TGA

**Figure 10:**
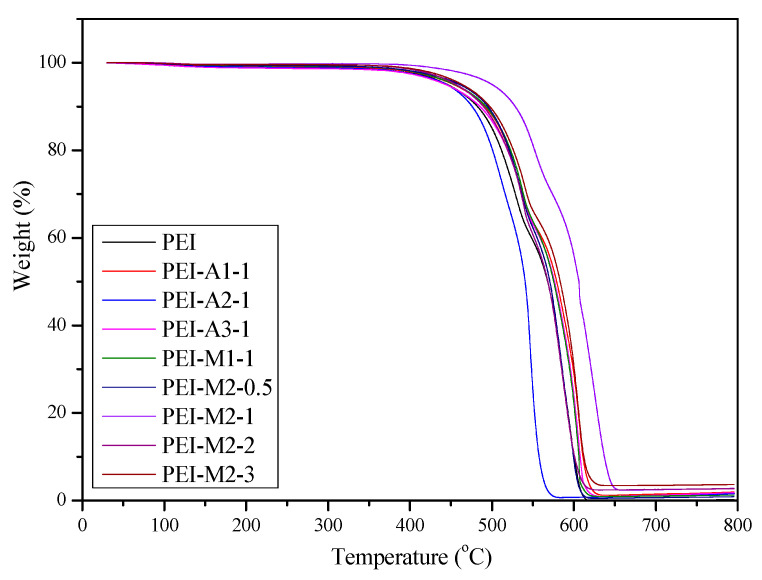
TGA curves for the PEI and its corresponding composite membranes containing five distinctive MSPs (A1, A1, A3, M1, and M2).

This section is divided into subheadings. It should provide a concise and precise description of the experimental results, their interpretation, as well as the experimental conclusions that could be drawn. The moisture absorption and decomposition temperature (T_5d_) of the PEI membranes were investigated with TGA, as shown in Figure 10 and Table 2. The experimental conditions were as follows: the operational temperature of the TGA study for the prepared membrane samples was run from 30 °C to 800 °C under atmospheric conditions at a heating rate of 10 °C/min.

First, the moisture absorption amount of the PEI and corresponding composite membranes was defined as the weight loss of the TGA curve at 200 °C. Therefore, the moisture absorption of the neat PEI membrane was estimated at ~0.73 wt-%, which may be due to the appearance of oxygen and nitrogen atoms on the surface of the neat PEI membrane. By introducing the hydrophilic A1, A1, and A3 MSPs, the moisture absorption of the composite membranes increased up to 0.95, 0.98, and 1.18 wt-%, respectively. On the other hand, by introducing the hydrophobic MSPs (M1 and M2), the moisture absorption of the composite membranes decreased to 0.69 and 0.52 wt-%, respectively. Moreover, by introducing the hydrophobic M2 MSP at four different feeding ratios of 0.5 wt-%, 1 wt-%, 2 wt-%, and 3 wt-% in the PEI membrane, the moisture absorption of the composite membranes was further decreased to 0.61, 0.52, 0.38, and 0.45 wt-%, respectively.

Second, the T_5d_ of the PEI and its corresponding composite membranes were also determined with the weight loss from the TGA curve at 5 wt-%. It should be noted that all PEI membrane samples exhibited T_5d_ 400 °C. First, the T_5d_ of the neat PEI membrane appeared at 445.8 °C. Moreover, the T_5d_ of the PEI membrane containing the hydrophilic MSPs (A1, A2, and A3) were located at 453.8, 445.0, and 443.9 °C, respectively.

On the other hand, the T_5d_ of the PEI membrane containing the hydrophobic MSPs of M1 and M2 were 461.0 and 470.4 °C, respectively. It indicated that the hydrophobic M2 MSP with a higher surface area might have stronger chemical bonds between the primary amine groups inside and outside the mesoporous silica channels with the dianhydride groups of TAHQ, simultaneously leading to higher mechanical strength and thermal decomposition temperature.

The T_5d_ of the PEI membrane containing the hydrophobic M2 MSP at four different feeding ratios of 0.5 wt-%, 1 wt-%, 2 wt-%, and 3 wt-% were found to be 424.8, 470.0, 486.6, and 463.9 °C, respectively. It implied that, up to the 2 wt-% loading of M2, a higher loading of M2 in the PEI membrane resulted in a higher thermal decomposition temperature of the composite membranes. However, the increase in the T_5d_ of the PEI composite membrane at 3 wt-% of M2 loading may be attributed to the following two reasons: (1) the M2 particle aggregated at higher loading of MSPs, resulting in decreased chemical bond formation between the -NH_2_ of M2 and the dianhydride of TAHQ or (2) the excessive amount of hydrophobic M2 particles existing in the solution of APAB and TAHQ retarded the polymerization reactions.

### 3.5. Discussion

The hydrophobic mesoporous silica powder prepared in this research experiment only used a methyl functional siloxane modifier for the hydrophobic modification. In future studies, non-fluorine-based modifier formulations could be considered as substitutions, such as siloxane with hydrophobic functional groups, including phenyl, vinyl, and ester groups could be adapted to modify the mesoporous silica. In addition, dispersibility could be studied and discussed by adjusting the particle size of the powder. The conditions of the sol-gel method could also be investigated in detail, such as varying the pH value of the synthesis environment. This could adjust the particle size of the powder, i.e., adjust the organic and inorganic phases, finding better compatibility. In addition, to achieve commercially practical applications, yield is also an important key factor because it is related to the cost of goods. The high specific surface area hydrophobic mesoporous silica powder M2 prepared in this study had a yield of approximately 25%, so it is bound to be optimized through conditions to improve the yield before it could be used in practical applications.

**Table 1:** Formulation and analytical data of the BET, CA, TGA, and EDS for the hydrophilic MSPs of A1, A2, and A3 as well as the hydrophobic MSPs of M1 and M2.

Sample Code	D-(-)-Fructose	TEOS	APTES (-NH_2_)	MTMS (-CH_3_)	BET			CA	TGA@200 °C	EDS (Atomic%)			
Unit	g	mmole	mmole	mmole	Surface Area (m^2^/g)	Pore Volume (cm^3^/g)	Average Pore Diameter (nm)	(°)	Moisture (%)	C	N	O	Si
A1	5.2	27	22.6	-	469 ± 12	0.26 ± 0.02	2.9 ± 0.2	-	4.15	49.25	0.80	36.59	13.36
A2	5.2	27	45.2	-	402 ± 21	0.25 ± 0.02	3.0 ± 0.2	-	4.24	26.41	1.20	51.55	20.84
A3	5.2	27	90.4	-	384 ± 14	0.21 ± 0.01	2.7 ± 0.1	-	7.68	34.05	1.60	41.53	22.83
M1	5.2	9	0.45	18	548 ± 12	0.94 ± 0.02	4.9 ± 0.1	127.6	0.58	-	-	-	-
M2	7.2	9	0.45	18	771 ± 14	0.86 ± 0.01	6.2 ± 0.1	127.2	0.45	-	-	-	-

All samples were prepared with 9 mL of ammonia and 100 mL of distilled water.

**Table 2:** Formulation and analytical data of the dielectric properties, mechanical strengths, thermal properties, moisture absorptions, and crystallinities of the PEI and its corresponding composite membranes containing A1, A2, A3, M1, and M2.

Sample Code	PEI		Silica		10 GHz		Tensile Strength	Elongation at Break	T_5d_	Moisture (TGA@200 °C)	Contact Angle	FWHM
	TAHQ	APAB										
Unit	(mmole)	(mmole)	Code	(wt-%)	D_k_	D_f_	MPa	%	°C	%	°	
PEI	20	20	-	-	3.27	0.007	11.26	1.48	445.8	0.73	74.6	3.2
PEI-A1-1	20	20	A1	1	3.43	0.011	4.03	2.15	453.8	0.95	83.7	3.4
PEI-A2-1	20	20	A2	1	3.46	0.012	4.80	2.52	445.0	0.98	80.9	3.5
PEI-A3-1	20	20	A3	1	3.92	0.015	5.48	2.75	443.9	1.18	61.5	3.5
PEI-M1-1	20	20	M1	1	3.24	0.009	-	-	461.0	0.69	83.5	3.3
PEI-M2-0.5	20	20	M2	0.5	3.24	0.007	15.91	1.37	424.8	0.61	81.1	3.3
PEI-M2-1	20	20	M2	1	3.18	0.008	16.23	1.20	470.4	0.52	84.3	3.3
PEI-M2-2	20	20	M2	2	2.97	0.008	18.95	1.19	486.6	0.38	88.5	3.4
PEI-M2-3	20	20	M2	3	2.93	0.009	6.68	1.65	463.9	0.45	93.8	3.5

## 4. Concluding Remarks

In this study, comparative studies of hydrophilic and hydrophobic MSPs on the dielectric properties of their derivative PEI composite membranes were performed. First of all, a series of hydrophilic and hydrophobic MSPs were synthesized via the base-catalyzed sol-gel process of TEOS, MTMS, and APTES at distinctive specific feeding ratios with the non-surfactant template of D-(-)-Fructose as the pore-forming agent. The prepared MSPs were characterized with FTIR, ^29^Si-NMR, and ^13^C NMR spectra, and EDS. The surface morphological images of the prepared hydrophilic MSPs modified with APTES and hydrophobic MSPs modified with MTMS were found to show spherical and irregular shapes, respectively, as identified by observing the SEM and TEM. For the study of BET, by increasing the APTES content of the hydrophilic MSPs, a trend of decreasing surface area and pore volume was found. On the other hand, by increasing the content of the pore-forming agent in the hydrophobic MSPs, the surface area and average pore diameter were both elevated. The contact angle of the hydrophilic MSPs (A1, A2, and A3) could not be detected, and the contact angle of the hydrophobic MSPs (M1 and M2) all measured at ~127°. The moisture absorptions of the A1, A2, A3, M1, and M2 MSPs were estimated at 4.15, 4.24, 7.68, 0.58, and 0.45 wt-%, respectively, as determined with TGA.

Subsequently, the MSPs were blended with the diamine of APAB, followed by the introduction of the dianhydride of TAHQ with mechanical stirring for 24 h. The obtained PEI2 composites were characterized with FTIR and XRD. It should be noted that the dielectric constant of the PEI composites was found to show an obvious trend: PEI containing hydrophilic MSP > PEI > PEI containing hydrophobic MSP. Moreover, the higher the loading of hydrophilic MSPs, the lower the dielectric properties. On the contrary, the higher the loading of the hydrophobic MSP, the better the dielectric properties. The mechanical strength and thermal stability of the PEI and its composite membranes were investigated with the tensile test and TGA, respectively. It should be noted that the PEI composite membranes containing hydrophilic MSPs and hydrophobic MSPs were found to exhibit weaker and stronger mechanical strengths, respectively. Moreover, the PEI composite containing hydrophilic and hydrophobic MSPs was also found to reveal lower and higher thermal decomposition temperatures, respectively. The PEI composite membranes containing hydrophilic and hydrophobic MSPs were found to exhibit higher and lower moisture absorption amounts, respectively.

## Data Availability

The data that support the findings of this study are available from the corresponding author, but restrictions apply to the availability of these data, which were used under license for the current study, and so are not publicly available. The data are, however, available from the authors upon reasonable request and with permission of the funding party, Ministry of Science and Technology, Taiwan, R.O.C.
